# An American Text-Book of Diseases of the Eye, Ear, Nose and Throat

**Published:** 1899-04

**Authors:** 


AN AMERICAN TEXT-BOOK OF DISEASES OF THE EYE, EAR, NOSE AND THROAT. Edited by G. E. de Schweinitz, A. M., M. D., Professor of Ophthalmology in the Jefferson Medical College, Philadelphia; Consulting Ophthalmologist to the Philadelphia Polyclinic; Ophthalmic Surgeon to the Philadelphia Hospital and to the Orthopedic Hospital and Infirmary for Nervous Diseases; B. Alex. Randall, M. A., M. D., Ph. D., Clinical Professor of Diseases of the Ear in the University of Pennsylvania; Professor of Diseases of the Ear in the Philadelphia Polyclinic; Ophthalmic and Aural Surgeon to the Methodist and Children's Hospital, Philadelphia. Illustrated with 766 engravings, 59 of them in colors. Philadelphia: W. B. Saunders, 925 Walnut Street. 1899. Price, cloth, $7 net; sheep or half morocco, $8 net.

This magnificent work, the joint product of more than fifty of the leading specialists of this country, and covering, as it does, three if not four of the leading specialties, is a distinct achievement of the medical profession of this country. It is doubtful if a more valuable book has been published in a decade. Its style is a model of direct simplicity, and this great special field is covered in a manner that will not prove tedious or dry to the busiest general practitioner. The book, including indices, covers 1251 pages, with more than 800 illustrations, over sixty of which are colored plates. The teaching is clinical to a large degree, and up to date, and covers many points of interest to the general practitioner, especially in diseases of the throat and air passages. Full advantage has been taken of all the latest discoveries in pathology and bacteriology. The mechanical execution seems to be perfect, and the price at which the work is offered is very reasonable. J.

